# Few-mode field quantization for multiple emitters

**DOI:** 10.1515/nanoph-2021-0795

**Published:** 2022-08-22

**Authors:** Mónica Sánchez-Barquilla, Francisco J. García-Vidal, Antonio I. Fernández-Domínguez, Johannes Feist

**Affiliations:** Departamento de Física Teórica de la Materia Condensada and Condensed Matter Physics Center (IFIMAC), Universidad Autónoma de Madrid, E-28049 Madrid, Spain; Institute of High Performance Computing, Agency for Science, Technology, and Research (A*STAR), Connexis, 138632 Singapore, Singapore

**Keywords:** few-mode quantization, hybrid cavities, multiple emitters, quantum nanophotonics, subwavelength cavity QED

## Abstract

The control of the interaction between quantum emitters using nanophotonic structures holds great promise for quantum technology applications, while its theoretical description for complex nanostructures is a highly demanding task as the electromagnetic (EM) modes form a high-dimensional continuum. We here introduce an approach that permits a quantized description of the full EM field through a small number of discrete modes. This extends the previous work in ref. (I. Medina, F. J. García-Vidal, A. I. Fernández-Domínguez, and J. Feist, “Few-mode field quantization of arbitrary electromagnetic spectral densities,” *Phys. Rev. Lett.*, vol. 126, p. 093601, 2021) to the case of an arbitrary number of emitters, without any restrictions on the emitter level structure or dipole operators. The low computational demand of this method makes it suitable for studying dynamics for a wide range of parameters. We illustrate the power of our approach for a system of three emitters placed within a hybrid metallodielectric photonic structure and show that excitation transfer is highly sensitive to the properties of the hybrid photonic–plasmonic modes.

## 1 Introduction

The control of photon-mediated interactions between quantum emitters has generated great interest over the last years, since it is essential for quantum technology applications such as quantum networking, quantum information, and quantum computation [1–5]. Nanophotonic devices with subwavelength light confinement are promising platforms to engineer such interactions, as the large confinement enables large emitter-photon coupling strengths and thus fast dynamics. At the same time, achieving strongly subwavelength confinement typically relies on the use of highly lossy constituents such as metallic nanoparticles with plasmonic resonances [6] and furthermore requires that the quantum emitters are brought close to the material surfaces. In these conditions, the EM mode spectrum typically contains a series of broad and overlapping resonances [7].

Quantizing the electromagnetic (EM) field in such systems is highly nontrivial, as losses cannot be neglected nor treated perturbatively, such that standard approaches of quantization fail [8, 9]. One powerful framework that overcomes these limitations is given by macroscopic quantum electrodynamics (QED) [10–18]. It provides a recipe for quantizing the medium-assisted EM field in material structures whose response is approximated through the macroscopic Maxwell’s equations, including dispersive and lossy materials. However, within this quantization scheme, the quantized EM field is described by an extremely large continuum of bosonic modes [18]. While this approach has proven hugely successful for treating problems where the EM modes are treated perturbatively or integrated out in some other way, it is not directly useful for applying cavity QED-like approaches in which the modes are treated as explicit degrees of freedom of the system.

In parallel to the work on macroscopic QED, there is a long history of approaches aiming to construct models for the EM or other environments based on a few lossy modes [19–25]. However, most of them cannot deal explicitly with material losses. The increasing interest in metallic and metallodielectric subwavelength cavity QED systems, in which highly lossy resonances act as effective cavity modes, has led to the development of several approaches that build on macroscopic QED and allow the construction of few-mode quantized models in which the full quantized EM field is approximately described through a (small) collection of discrete quantized modes. One approach relies on quasinormal mode theory for Maxwell’s equations [26–28]. It is based on forming superpositions of the modes of macroscopic QED that correspond to the quasinormal modes (resonances) of the material structure and then performing appropriate approximations to obtain the energies, decay rates, and coherent and incoherent interactions of these modes [29–31]. This approach is powerful but relies on being able to select just a few quasinormal modes of the system. In the case of nanometric-sized metallic structures, a similar quantization strategy has been used, but greatly simplified through the quasi-static description of sub-wavelength-confined plasmonic fields, which allows the spectral density to be written in terms of independent Lorentzians, thus allowing its quantization in terms of noninteracting lossy modes [7, 32], [33], [34]. An alternative approach is obtained by exploiting the fact that, due to its linearity, a system of harmonic oscillators (such as EM modes) is fully determined by its linear response to the quantum emitter, encoded in the so-called spectral density. This viewpoint is inspired by the field of open quantum systems and unlocks the possibility to use the many tools of that field [35–40]. In particular, this includes the idea to construct a model environment that shows the same response as the real one but is (significantly) easier to solve than the original problem. It has recently been shown that a discrete collection of interacting modes coupled to fully Markovian background baths provides exactly such a model with sufficient flexibility to reproduce the complex response of typical nanophotonic systems [1], while leading to a relatively easily solvable cavity QED-like few-mode model. This is achieved by explicitly enforcing Markovianity of the background baths in the model construction, which is not easily obtained without approximations when the modes are constructed from a partitioning of the underlying EM problem [22, 25]. The model thus circumvents the problem of finding a direct simplification of macroscopic QED to a few-mode model and replaces it by a fitting procedure for which the degree of convergence can be checked by comparing the EM and model spectral densities. Another important advantage of this model lies in the fact that often just one or a few model modes are enough to accurately represent peaks in the spectral density that arise due to the collective action of many overlapping quasi-degenerate physical resonances of the system. In contrast, in approaches based on quasinormal modes, all physical resonances must be included in the description, and achieving convergence of the spectral density is challenging. Such a situation is often encountered in the so-called pseudomodes (unrelated to the concept of pseudomodes used in the literature on quantization of lossy modes [21]) in plasmonic systems, which arise due to the collective response of high-k modes in planar systems [41] or high-order multipoles in spherical ones [32].

While the model developed in ref. [1] can treat a wide range of nanophotonic structures, in the formulation presented therein, it is only suitable for situations where only a single emitter is present in the system and all considered emitter dipole transitions are co-aligned. In the present article, we lift these restrictions and extend the approach to a collection of emitters with arbitrary orientations of the transition dipole moments. We achieve this by first generalizing the definition of the spectral density to the case of several light–matter interaction operators [42–44]. The spectral density *J*(*ω*), which is normally a scalar function that fully characterizes the interaction between a quantum system and a bath mediated by a single interaction operator [39, 45], then becomes an *M* × *M* matrix-valued function. Here, *M* is the number of distinct interaction operators that are treated (*M* = 3*M*_*e*_ for *M*_*e*_ dipolar emitters with all three possible dipole orientations taken into account for each emitter). We then extend the few-mode quantization approach presented in ref. [1] to this case. We show that also in this case, a simple fitting procedure leads to a few-mode quantization of generalized spectral densities for several emitters placed at different positions.

We then apply the approach to study energy transfer between emitters for three different situations: (i) transfer of a single excitation from a coherent superposition of two emitters to a third one; (ii) transfer of a single excitation from one emitter to another, mediated by the third one; and (iii) excitation transfer to a third emitter when the other two emitters are initially excited. Our method is able to calculate the dynamics for these different examples at low computational cost. They show that the use of metallodielectric structures allows great control in the population transfer between emitters close to resonance to a hybrid mode, with slight changes in the emitter parameters inducing qualitatively different dynamics.

## 2 Theory

We start by discussing a general model consisting of a matter part (which can represent multiple emitters) linearly coupled to a collection of bosonic modes (which will later represent the medium-assisted EM field). We set *ℏ* = 1 here and in the following, and write the Hamiltonian as

(1) $$H=H_{\text{mat}}+{\vec{A}}^{†T}H\vec{A}+\left({\vec{V}}^{T}\cdot M\cdot \vec{A}+H.c.\right),$$

where *H*_mat_ describes the matter (the emitters), $\vec{A}={\left(a_{1},a_{2},\ldots ,a_{\alpha },\ldots \right)}^{T}$ collects all EM modes, and $\vec{V}={\left(V_{1},V_{2},\ldots ,V_{n},\ldots \right)}^{T}$ collects the emitter operators describing the interaction with the bosonic modes. In the case considered below, $\vec{V}$ will contain the dipole operators (up to three per emitter if all polarizations have to be taken into account). The properties of the bosonic environment are thus fully encoded in the matrices **H** (size *N*_*a*_ × *N*_*a*_) and **M** (size *M* × *N*_*a*_), where *N*_*a*_ is the number of bosonic modes and *M* is the number of matter interaction operators. We note that for simplicity of notation, we discuss a formally discretized bosonic bath and will perform the continuum limit *N*_*a*_ → ∞ when required.

We now define the generalized spectral density associated to the bosonic environment in Eq. (1) as

(2) $$J\left(\omega \right)=\underset{\epsilon \to 0}{\lim}\frac{1}{\pi }MIm\left[\frac{1}{H-\omega -i\epsilon }\right]M^{†}.$$

This definition is a straightforward extension of the single-emitter spectral density to the case of multiple light–matter interaction operators, obtained by replacing a 1 × *N*_*a*_ vector of light–matter coupling elements by the *M* × *N*_*a*_ matrix **M**, and has been previously obtained in the context of the Wigner–Weisskopf problem (i.e., within the single-excitation subspace) [42–44]. We note that it is not *a priori* clear whether J(ω) encodes the full information about the environment that the emitters interact with (as is well known for the case of 1D-spectral densities). Below, we show that this is indeed the case. The generalized spectral density is an *M* × *M* matrix-valued function of frequency and fulfills $J{\left(\omega \right)}^{†}=J\left(\omega \right)$ . We note that when the emitters are approximated as two-level systems, the transition dipole moments are usually included in **M**, such that the interaction operators *V*_*n*_ become unitless and J(ω) has units of frequency [42].

If **H** is diagonal, *H*_*αβ*_ = *ω*_*α*_ *δ*_*αβ*_, we can use the Sokhotski–Plemelj formula $\lim_{\epsilon \to 0}\frac{1}{\omega^{\prime }-\omega -i\epsilon }=P\frac{1}{\omega^{\prime }-\omega }+i\pi \delta \left(\omega^{\prime }-\omega \right)$ to get

(3) $$J_{nm}\left(\omega \right)=\underset{\alpha }{\sum }M_{n\alpha }M_{m\alpha }^{*}\delta \left(\omega -\omega_{\alpha }\right),$$

which is a form where the relation to conventional single-emitter spectral densities *J*(*ω*) = *∑*_*α*_|*M*_*α*_|^2^ *δ*(*ω* − *ω*_*α*_) appears even more clearly.

To connect the general Hamiltonian Eq. (1) to the physical system we are interested in (a collection of emitters interacting with the EM field supported by a material structure), we use the framework of macroscopic QED. The Hamiltonian in the multipolar coupling scheme (Power–Zienau–Woolley picture) and within the dipole approximation can then be written as

(4) $$\begin{array}{ll} H & =\underset{\lambda }{\sum }\int d^{3}r\overset{\infty }{\underset{0}{\int }}d\omega \;\omega {\hat{f}}_{\lambda }^{†}\left(r,\omega \right){\hat{f}}_{\lambda }\left(r,\omega \right)\\ & \;+\underset{k}{\sum }H_{k}-\underset{k}{\sum }\mu_{k}\cdot \hat{E}\left(r_{k}\right), \end{array}$$

where *λ* = {e, *m*} labels the electric and magnetic contributions, *M* is the number of emitters, ${\hat{f}}_{\lambda }\left(r,\omega \right)$ and ${\hat{f}}_{\lambda }^{†}\left(r,\omega \right)$ are the bosonic annihilation and creation operators of the medium-assisted field, and *H*_*k*_ and *μ*_*k*_ are the bare Hamiltonian and dipole operator of emitter *k*. The electric field operator is given by

(5) $$\hat{E}\left(r\right)=\underset{\lambda }{\sum }\int d^{3}r^{\prime }\overset{\infty }{\underset{0}{\int }}d\omega G_{\lambda }\left(r,r^{\prime },\omega \right)\cdot {\hat{f}}_{\lambda }\left(r^{\prime },\omega \right)+\text{H.c.},$$

where **G**_*λ*_(**r**, **r**′, *ω*) are the electric and magnetic Green’s functions, given by [15]

(6) $$G_{e}\left(r,r^{\prime },\omega \right)=i\frac{\omega^{2}}{c^{2}}\sqrt{\frac{\hbar }{\pi \epsilon_{0}}Im\epsilon \left(r,\omega \right)}G\left(r,r^{\prime },\omega \right),$$

(7) $$G_{m}\left(r,r^{\prime },\omega \right)=-i\frac{\omega }{c}\sqrt{\frac{\hbar }{\pi \epsilon_{0}}\frac{Im\mu \left(r,\omega \right)}{|\mu \left(r,\omega \right){|}^{2}}}G\left(r,r^{\prime },\omega \right)\times \overset{\leftarrow }{\nabla^{\prime }}.$$

In this approach, retardation effects are fully included and encoded in the EM Green’s function. This Hamiltonian can be rewritten in the form of Eq. (1) by formally discretizing space and frequency and defining $a_{\alpha }=n_{\alpha }\cdot {\hat{f}}_{\lambda_{\alpha }}\left(r_{\alpha },\omega_{\alpha }\right)$ , with a combined mode index *α* ≡ (*λ*_*α*_, **r**_*α*_, *ω*_*α*_, **n**_*α*_), where $n_{\alpha }\in \left\{\hat{x},\hat{y},\hat{z}\right\}$ . Furthermore, the interaction operators $V_{n}=n_{n}\cdot \mu_{k_{n}}$ are determined by a combined index *n* ≡ (*k*_*n*_, **n**_*n*_), where the unit vectors **n**_*n*_ give the (up to three) dipole directions taken into account for each emitter. This way, we can identify *H*_*αβ*_ = *δ*_*αβ*_ *ω*_*α*_ and $M_{n\alpha }=-n_{n}\cdot G_{\lambda_{\alpha }}\left(r_{n},r_{\alpha },\omega_{\alpha }\right)\cdot n_{\alpha }$ .

Inserting the expression for *M*_*nα*_ in Eq. (3), taking the continuum limit (i.e., replacing the sum over *α* with the corresponding sums and integrals), and using the Green’s function integral identity [15]

(8) $$\begin{array}{ll} & \underset{\lambda }{\sum }\;\int \;d^{3}sG_{\lambda }\left(r,s,\omega \right)\cdot G_{\lambda }^{*T}\left(r^{\prime },s,\omega \right)\\ & \;=\frac{\omega^{2}}{\pi \epsilon_{0}c^{2}}ImG\left(r,r^{\prime },\omega \right) \end{array}$$

leads to

(9) $$J_{nm}\left(\omega \right)=\frac{\omega^{2}}{\pi \epsilon_{0}c^{2}}n_{n}\cdot ImG\left(r_{n},r_{m},\omega \right)\cdot n_{m},$$

where **G** is the conventional dyadic Green’s function. The generalized spectral density of an EM environment is thus seen to be directly related to the so-called cross density of states used to characterize the spatial coherence of photonic systems [46, 47]. The diagonal elements of J(ω) are equal to the “conventional” spectral density for a single transition up to the square of the dipole transition matrix element, $\mu_{n}^{2}J_{nn}\left(\omega \right)=J_{n}\left(\omega \right)$ . From Eq. (9), it can be seen that J(ω) is a real and symmetric matrix for any frequency *ω*, and due to the properties of the EM dyadic Green’s function, it is also positive definite. This means that it can be decomposed in the form $J\left(\omega \right)=g\left(\omega \right)g{\left(\omega \right)}^{†}$ , where **g**(*ω*) can be chosen real and is unique up to unitary transformations **g**′(*ω*) = **g**(*ω*)**U**(*ω*). We now note that **g**(*ω*) is exactly the coupling matrix that appears in the expansion of the multi-emitter problem using emitter-centered modes in macroscopic QED [18]. As discussed and demonstrated in that reference, this quantity indeed encodes the full information about the EM environment, and consequently, so does J(ω) . This means that two different systems with the same J(ω) are indistinguishable from the point of view of the emitters. We note in passing that J(ω) is in fact a more fundamental quantity than **g**(*ω*), as it does not depend on an arbitrary choice of basis.

We now extend the model presented in ref. [1] in order to obtain an effective few-mode description of the multi-emitter problem. As discussed there, the idea is to find a model system that is equivalent to the actual EM environment but has a structure that facilitates its numerical solution and the interpretation of the resulting dynamics. We again introduce a discrete set of *N* mutually coupled discrete EM modes *a*_*i*_, each of which is coupled to an independent bath of “background” modes *b*_*i*,Ω_ with frequency-independent coupling determined by $\nu_{i}=\sqrt{\kappa_{i}/\left(2\pi \right)}$ and also to the emitter interaction operator *V*_*n*_ with coupling strength *g*_*ni*_. The background bath modes are not directly coupled to the emitters. The model Hamiltonian is then given by $H=H_{S}+H_{B}$ , where

(10a) $$H_{S}=\underset{n}{\sum }H_{n}+\underset{i,j}{\sum }\omega_{ij}a_{i}^{†}a_{j}+\underset{n,i}{\sum }V_{n}g_{ni}\left(a_{i}+a_{i}^{†}\right),$$

(10b) $$H_{B}=\underset{i}{\sum }\overset{\infty }{\underset{-\infty }{\int }}\left[\Omega b_{i,\Omega }^{†}b_{i,\Omega }+\nu_{i}\left(b_{i,\Omega }^{†}a_{i}+b_{i,\Omega }a_{i}^{†}\right)\right]d\Omega .$$

Since the coupling of the discrete modes to the background baths is spectrally flat and extends over the full real axis, it is perfectly Markovian and furthermore does not induce energy shifts on the discrete modes. The dynamics of the system are then equivalently described [39, 48] by the Lindblad master equation

(11) $$\dot{\rho }=-i\left[H_{S},\rho \right]+\underset{i}{\sum }\kappa_{i}L_{a_{i}}\left[\rho \right],$$

where *ρ* is the system density matrix and $L_{O}\left[\rho \right]=O\rho O^{†}-\frac{1}{2}\left\{O^{†}O,\rho \right\}$ is a Lindblad dissipator. We note here that since the frequency integrals in Eq. (10b) extend over the full real axis, the model spectral density is not necessarily zero at negative frequencies. In contrast, the EM spectral density at zero temperature is only nonzero for positive frequencies. Depending on the physical processes under study, some care has to be taken to ensure that nonzero negative frequency components do not induce artificial effects in the dynamics (which tends to happen when counterrotating terms are important in the light–matter coupling, such as in the limit of ultrastrong coupling [49]).

The model Hamiltonian H can also be rewritten in the form of Eq. (1). To do so, we formally discretize the bath continua, with *N*_*b*_ modes for each continuum, such that there are *N*_*a*_ = *N*(*N*_*b*_ + 1) bosonic modes, given by $\vec{A}={\left(a_{1},\ldots ,a_{N},b_{1,\Omega_{1}},\ldots ,b_{1,\Omega_{N_{b}}},b_{2,\Omega_{1}},\ldots ,b_{N,\Omega_{N_{b}}}\right)}^{T}$ . In this form, **H** is not diagonal but consists of the block matrix *ω*_*ij*_ in the top left, a series of diagonal blocks for each continuum, and constant off-diagonal coupling elements between all modes of a block and the discrete mode associated to it. Finally, **M** is an *M* × *N*(*N*_*b*_ + 1) matrix in block form, $M=\left(\begin{array}{cc} g & 0 \end{array}\right)$ , where **g** is the real *M* × *N* matrix containing the coupling elements *g*_*ni*_.

A compact form of the generalized spectral density of the model system can be obtained from Eq. (2), either by explicit diagonalization of **H** using the Lippmann–Schwinger formalism as in the supplemental material of ref. [1] (see [25] for an overview of the method) or by following the approach of ref. [50]. The resulting expression is

(12) $$J_{mod}\left(\omega \right)=\frac{1}{\pi }gIm\left[\frac{1}{\tilde{H}-\omega }\right]g^{T},$$

where $\tilde{H}$ is a complex symmetric *N* × *N* matrix with elements given by ${\tilde{H}}_{ij}=\omega_{ij}-\frac{i}{2}\kappa_{i}\delta_{ij}$ . When $\tilde{H}$ is diagonalizable, $\tilde{H}=V\tilde{\Omega }V^{T}$ , where $\tilde{\Omega }$ is a diagonal matrix containing the (complex) eigenvalues and **V** is a complex orthogonal (not unitary) matrix, **V** ^*T*^ **V** = **1**, this expression can be rewritten as a sum over resonances,

(13) $$J_{mod}\left(\omega \right)=\frac{1}{\pi }Im\left[\tilde{g}\frac{1}{\tilde{\Omega }-\omega }{\tilde{g}}^{T}\right],$$

where $\tilde{g}=gV$ . This diagonal form facilitates the classification of multi-mode effects [51]. Furthermore, this form makes clear that the complex eigenvalues of $\tilde{H}$ are poles of the model spectral density, $J_{mod}\left(\omega \right)$ . This implies that when the physical spectral density (determined by the EM Green’s function) is dominated by a clear set of resonances, the number of discrete modes needed to approximate it closely can be quite small, with about one mode per peak. In this limit and for a sufficiently converged fit, the poles of the model spectral density will thus coincide closely with the dominant poles of the classical Green’s function, which correspond to the quasinormal modes that contribute most strongly to the Green’s function. We also note here that although we have assumed that the EM modes are initially in the vacuum state, nonzero temperature could be implemented by replacing the spectral density by the (temperature-dependent) bath noise-power spectrum [52], while again using the vacuum state as the initial state of the “temperature-adjusted” bath. Furthermore, (classical) external driving fields can also be straightforwardly included [18, 53]. Finally, it has recently been shown that quasinormal mode quantization can also be performed in gain media [30], and a similar extension of the current approach (which is restricted to absorbing materials) is probably possible.

As in the single-emitter case, the parameters in Eq. (12) can be adjusted to best reproduce the generalized spectral density Eq. (9) calculated from the dyadic EM Green’s function and thus parametrize the model Hamiltonian H . We note that a recent article provided convergence guarantees for such a fit (for spectral densities fulfilling some specific assumptions) even for the case of noninteracting modes (i.e., where *ω*_*ij*_ = *ω*_*ii*_ *δ*_*ij*_) [54]. However, the proof given in ref. [54] relies on the limit of vanishing mode decay rates, which essentially amounts to a direct discretization of the continuum. While this limit can be represented within our approach, the resulting model is not useful in practice if the number of modes becomes too high. For the system and parameter regime we study here, excellent convergence is reached with relatively small numbers of modes (around one per peak, which is much smaller than the number of quasinormal modes required to reproduce the full spectral density). However, it is well known that, e.g., in the ultrastrong-coupling limit, a single mode with a Lindblad decay term leads to unphysical effects [23, 55], [56], [57], such that the mapping of physical resonances to discrete modes in our model can be expected to break down. The practical utility of the approach will thus depend on the specifics of the spectral density, the range of relevant frequencies, and the physical processes of interest.

In the current work, the fit procedure used the optimization routines included in SciPy [58]. The overall fit was done in steps, i.e., “emitter by emitter,” where first the (diagonal) spectral density of one emitter was fitted and then used as the initial guess for the fit of two emitters and their interactions. This two-emitter fit was then in turn used as part of the initial guess for all three emitters and their interactions. We emphasize here that, in order to give a correct description of the whole system, the full matrix-valued J(ω) including the off-diagonal components must be reproduced. The symmetry of J(ω) means that this corresponds to a simultaneous fit of *M*(*M* + 1)/2 real-valued functions. The off-diagonal elements of the generalized spectral density, which encode the interaction between the emitters mediated by the EM fields, can have negative values, while the diagonal elements, which correspond to the “conventional” spectral densities, are non-negative, i.e., *J*_*nn*_(*ω*) ≥ 0 for all *ω*. We note that the spectral density is fully determined by the position of the emitters, with no restriction on the emitter properties. In particular, this approach is not restricted to two-level systems. However, if only one or two dipole orientations are relevant for the transitions of any emitter, the number of necessary interaction operators and thus the dimensionality of J(ω) can be reduced.

For completeness, we note that the number of free parameters in the diagonal form given in Eq. (13) is typically significantly smaller than in the nondiagonal form given in Eq. (12). In the nondiagonal form, the number of real parameters needed is $\frac{1}{2}N\left(N+1\right)$ for *ω*_*ij*_, *N* for *κ*_*i*_, and *NM* for *g*_*ni*_, giving a total of $\frac{1}{2}N\left(N+2M+3\right)$ free real parameters. In the diagonal form, there are *N* complex parameters ${\tilde{\Omega }}_{i}$ , while $\tilde{g}$ has *NM* complex parameters that are restricted by up to $\frac{1}{2}M\left(M+1\right)$ relations since $\tilde{g}{\tilde{g}}^{T}=gg^{T}$ must be purely real, giving $\frac{1}{2}\left(4N-M\right)\left(M+1\right)$ real parameters (when *N* > *M*). It would thus seem that this form is more convenient for fitting than the nondiagonal form. However, this turns out not to be the case [59]: first, in this form, it is not straightforward to enforce that the fit parameters correspond to a physical system where $J_{mod}\left(\omega \right)$ is real positive semidefinite for all frequencies. Second, even when that constraint is achieved, implementation of the dynamics through a Lindblad master equation (which as discussed above is the final goal of this approach) requires a form in which the imaginary part of the complex symmetric matrix $\tilde{H}$ is negative semidefinite, while **g** is real. This would require an algorithm that finds a complex orthogonal matrix **V**, which “undiagonalizes” the system and can be used to obtain physical $\tilde{H}=V\tilde{\Omega }V^{T}$ and $g=\tilde{g}V^{T}$ for any given $\tilde{\Omega }$ and $\tilde{g}$ . To the best of our knowledge, no algorithm that achieves this has been found [59, 60]. It is thus more straightforward to directly use the nondiagonal form Eq. (12) when fitting.

## 3 Results

**Figure 1:**
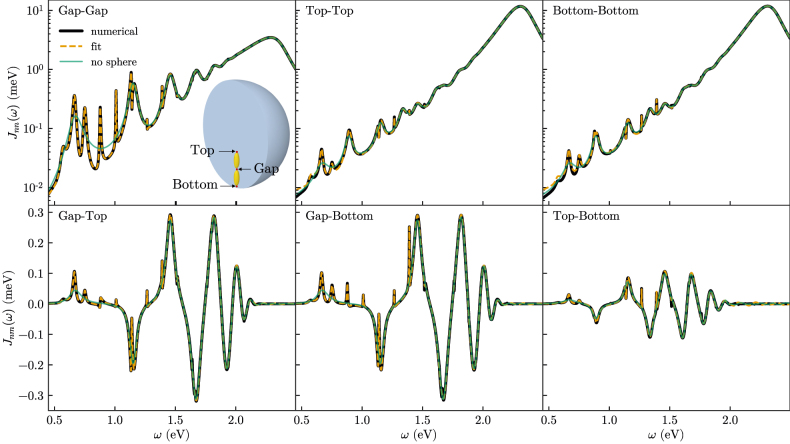
Generalized spectral densities for *z*-oriented emitters at Gap, Top and Bottom positions (thick black line), fitted model spectral density (orange line), and spectral density when the microsphere is replaced by a dielectric background (green line). Inset: Sketch of the system consisting of a silver dimer nanoantenna embedded in a dielectric microsphere (with the same dimensions as in ref. [1]). The red dots show the position of each emitter.

To illustrate the generalization to the multiple emitter case, we consider the same physical setup as in ref. [1], see the inset in Figure 1. It consists of two silver nanoparticles (ellipsoids with long axis of 120 nm and short axis of 40 nm), separated by a 3 nm gap, and embedded in a dielectric **Gallium phosphide** (**GaP,** *ϵ*_sph_ = 9) nanosphere of 600 nm radius, with the rods substantially displaced from the center of the sphere. We consider three two-level emitters placed in different positions, indicated by red dots in the inset in Figure 1. We will refer to the emitter positions as: (i) Gap (in the center between the rods), (ii) Top (1.5 nm above the upper rod), and (iii) Bottom (1.5 nm below the lower rod). The generalized spectral density then is characterized by six independent functions (three diagonal and three off-diagonal). Since we consider two-level quantum emitters with a single dipole transition, we include the transition dipole moments within the spectral density for simplicity, $J_{nm}\left(\omega \right)=\mu_{n}\mu_{m}J_{nm}\left(\omega \right)$ , with values given by *μ*_Top_ = *μ*_Bottom_ = 0.55 e nm and *μ*_Gap_ = 0.257 e nm. The interaction operators are then just $V_{n}=\sigma_{n}^{+}+\sigma_{n}^{-}$ . The physical system, as well as the emitter dipole moments, have been chosen so that the validity of the model and the potential of these structures can be illustrated. Nonetheless, the used parameters are within a realistic range, as similar dipole moments have been shown in quantum dots [61] and in (short) J-aggregates [62] that would fit within the gap. Additionally, similar couplings, and therefore similar effects as the ones studied in this article, could be obtained by decreasing both the dipole moment and the gap size. Figure 1 shows the generalized spectral density (black lines) obtained from classical simulations performed with the Maxwell equations solver implemented in the COMSOL Multiphysics^®^ software [63]. The first row shows the diagonal functions (in logarithmic scale), i.e., *J*_*nn*_(*ω*), which correspond to the “conventional” spectral densities, while the second row shows the off-diagonal functions, *J*_12_(*ω*), *J*_13_(*ω*), and *J*_23_(*ω*), which encode the field-mediated interaction between the emitters. As Top and Bottom are symmetric positions with respect to the plasmonic nanoparticles, both their diagonal elements and their interaction with the emitter in Gap behave quite similarly, with slight differences due to the nonsymmetric placement of the dielectric sphere.

In its most general form, there are no restrictions on the form of $\tilde{H}$ apart from symmetry. However, it is possible to choose any desired structure, e.g., inspired by the physical structure of the problem, to restrict the number of free parameters. In ref. [59], we recently showed that for the one-emitter case, it is generally sufficient to use a chain form with only next-nearest neighbor coupling between the modes (i.e., ${\tilde{H}}_{ij}=0$ if |*i* − *j*| >2), although this choice makes it more challenging to obtain converged fits. We here instead choose a block-diagonal form,

(14) $$\tilde{H}=\left(\begin{array}{ccc} {\tilde{H}}_{1} & 0 & 0\\ 0 & {\tilde{H}}_{2} & 0\\ 0 & 0 & {\tilde{H}}_{3} \end{array}\right),$$

where ${\tilde{H}}_{i}$ are full *N*_*i*_ × *N*_*i*_ matrices. This form allows to significantly decrease the number of parameters while still giving a good fit for the spectral density. The physical reasoning behind this election is that there are many independent modes in the system that do not interfere significantly (e.g., the high-order “pseudomodes” coupling to each emitter [32]), and it is thus not necessary to allow arbitrary couplings between all modes. In the present case, the size of each block was chosen as *N*_*i*_ = 14, giving a total of *N* = ∑_*i*_ *N*_*i*_ = 42 modes. We note that while this is not a small number per se, this number of modes is enough to represent the complex EM environment over the full spectral range shown in Figure 1, which contains many physical resonances. If only a restricted frequency range is of interest, the fit could be performed only within that range, or alternatively, adiabatic elimination of highly detuned modes could be performed after the fitting.

The orange lines in Figure 1 show the model spectral density obtained after fitting. Despite the complexity of the structure and the spectral densities, the fit is well converged with a relatively small number of modes. The small differences to the numerical spectral density visible at low frequencies for the diagonal functions and at high frequencies for the nondiagonal ones could be reduced by increasing the number of modes employed in the fit but were found not to affect the dynamics studied in this work.

In Figure 1, we additionally show the spectral density corresponding to the two plasmonic nanoparticles when the sphere is replaced by an infinite GaP background medium (green lines). In this case, Top and Bottom are completely equivalent positions, and their spectral densities are identical. Additionally, this change removes the Mie resonances supported by the sphere, such that the spectral density is overall much simpler and contains fewer peaks. In particular, there are no visible interference structures, and the spectral density corresponds to a series of broad but mostly independent modes. The fitting procedure then converges much more easily and achieves even better agreement with the numerical results, with $|J_{mod}\left(\omega \right)-J\left(\omega \right)|<0.015\;$ meV over the full spectral range of Figure 1 using *N* = 30 modes. As the fit is visually indistinguishable from the exact spectral density, it is not shown separately in Figure 1.

**Figure 2:**
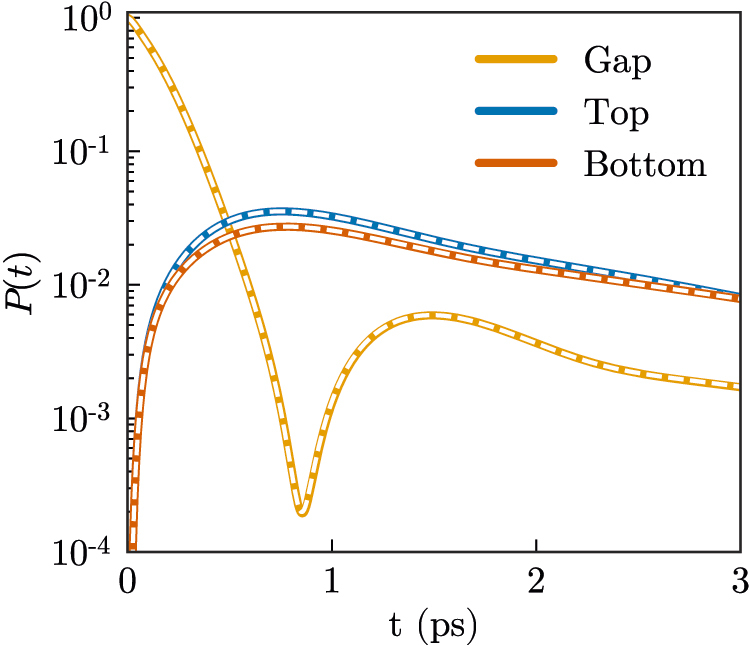
Population of Gap (orange), Top (blue) and Bottom (red) emitters within the hybrid metallodielectric system shown in Figure 1 when Gap is initially (*t* = 0) excited, while Top and Bottom are in their ground states. The colored lines correspond to a direct discretization of the photon continua in frequency, while the white dashed lines correspond to the dynamics predicted by the model.

In order to benchmark the method, Figure 2 shows the dynamics of all emitters for the Wigner–Weisskopf problem of spontaneous emission for the Gap emitter, i.e., when it is initially in the excited state, while the Top and Bottom emitters are in the ground state and all the EM field is in the vacuum, so that $|\psi \left(t=0\right)〉=\sigma_{\text{Gap}}^{+}|0〉$ . We choose emitters with frequencies close to the lowest-energy hybrid modes at ≈1.14 eV. In this case, *ω*_Top_ = *ω*_Bottom_ = 1.14 eV, while *ω*_Gap_ = 1.143 eV, so that the Lamb-shifted emitters are close to resonance (as discussed below). The dynamics predicted by our model (white dashed lines in Figure 2) are compared to the ones given by a direct discretization of the Hamiltonian based on emitted-centered modes (Eq. (21) in ref. [18]). Once the fit of the spectral density is converged with sufficient accuracy, there is an almost exact agreement of the emitter dynamics. Although the strength of the coupling is not large enough to unambiguously reach the strong coupling regime, there are clear oscillations in the population of the Gap emitter, which are perfectly reproduced by the few-mode model, demonstrating explicitly that the model is not restricted to weak coupling situations.

**Figure 3:**
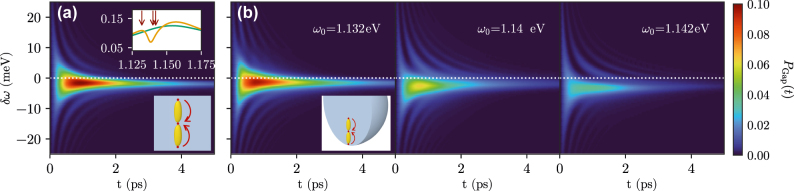
Population transfer to Gap when Top and Bottom are fixed at a frequency *ω*_0_ and Gap is detuned from that frequency. The initial state *ψ*_0_ is a superposition of Top and Bottom excited, as shown in both sketches in subplots (a) for the dielectric background and (b) for the dielectric sphere, where three different frequencies are considered (shown in each subplot). The upper inset in subplot (a) shows the spectral density of the Gap emitter around the hybrid mode for the dielectric sphere (orange line) and the dielectric background (green line).

We now study the energy transfer dynamics between the emitters, with a focus on how it is influenced by the formation of hybrid modes. The upper inset in Figure 3(a) shows the spectral density of the Gap emitter in that frequency range, both for the hybrid metallodielectric cavity (orange line) and for the plasmonic dimer embedded in an infinite dielectric medium (green line). For the hybrid cavity, there is significant mode hybridization and destructive interference around that frequency, while for the bare dimer, only a single broad resonance peak appears. We note here that within this model, the system is assumed to be at zero temperature, so that pure dephasing is neglected. Furthermore, while we do not explicitly include external driving, this could be done straightforwardly [18, 53]. Similarly, we only study the emitter dynamics, but the emitted EM field could be explicitly obtained using the approach shown in [1]. For completeness, we mention that high-order correlations of the EM fields are somewhat cumbersome to obtain within that approach.

We first investigate population transfer from a coherent superposition of the Top and Bottom emitters to the Gap emitter, as schematically shown in the lower insets of Figure 3. To be precise, we calculate the dynamics for an initial state $|\psi_{0}〉=\frac{1}{\sqrt{2}}\left(\sigma_{\text{Top}}^{+}+\sigma_{\text{Bottom}}^{+}\right)|0〉$ . We fix the frequencies of the Top and Bottom emitters to be equal, *ω*_Top_ = *ω*_Bottom_ = *ω*_0_ and vary the frequency of the Gap emitter, *ω*_Gap_ = *ω*_0_ + *δω*. The population $P_{\text{Gap}}\left(t\right)=\left\langle \sigma_{\text{Gap}}^{+}\sigma_{\text{Gap}}^{-}\right\rangle$ as a function of time and *δω* is shown for three distinct values of *ω*_0_, indicated by the dark red arrows in the upper inset of Figure 3. Panel (a) corresponds to the case of a dielectric background, for which we only consider *ω*_0_ = 1.142 eV (the results for the other two values are very similar due to the broad nature of the peak). We find significant excitation of the Gap emitter for a narrow range of frequencies, which however does not coincide with *δω* = 0 as could be naively expected. This is due to the fact that the EM modes induce a significant Lamb shift on the emitters, which is larger for the Top and Bottom emitters due to their higher dipole moments (even though the EM mode density at the Gap position is higher due to the interaction with both ellipsoids). Their effective frequencies are thus lowered more than that of the Gap emitter, and resonant energy transfer is only possible when the Gap emitter is detuned to a slightly lower frequency, *δω* ≈ −3 meV.

In panel (b), we explore the situation for the hybrid cavity, where the peak splits due to interaction between the Mie resonances of the microsphere and the plasmonic dimer modes. Since the spectral density here has significantly more structure, we explore the energy transfer for three values of *ω*_0_: 1.132 eV, 1.14 eV, and 1.142 eV. As shown in Figure 3(b), these small changes in frequency have a significant effect on the efficiency of energy transfer even when the detuning is optimized to compensate for the difference in Lamb shifts. In particular, the maximum population reaching the Gap emitter is decreased by a factor of more than two when changing *ω*_0_ by just 10 meV. This demonstrates both the sensitivity of energy transfer at the nanoscale to the details of the EM environment and the large degree of control that hybrid metallodielectric structures offer for influencing emitter dynamics. We also note that even though the emitters are not in the regime of strong light–matter coupling (no Rabi oscillations are visible on resonance), the effects observed here could not be reproduced with traditional methods based on the weak-coupling approximation [64–67]. In that approach, the EM environment is traced out fully, with the real and imaginary parts of the Green’s function giving coherent and incoherent interactions between emitters (with the diagonal parts corresponding to Lamb shifts and decay rates). However, that approach is only valid when the Green’s function is approximately constant over the frequency range spanned by the emitters (and the emitters are two-level systems characterized by a single frequency). It furthermore evaluates the Green’s function at the bare emitter frequencies and thus also becomes invalid if the Green’s function varies significantly over a frequency range comparable to the EM-induced shifts. For highly structured spectral densities as in the present case, this can be a significant source of error. We additionally note that the effects discussed here are not correctly represented either when only the EM modes close to resonance with the emitters are taken into account, as the Lamb shift is dominated by off-resonant contributions. Of course, these considerations do not imply that it would be in principle impossible to obtain a simpler approximate master equation that also describes the dynamics for any specific situation accurately. However, one important advantage of our current approach is that it is general and expected to work for any combination of emitter structures, energies, and orientations, i.e., it does not rely on any specific assumptions about the EM mode structure or emitter properties.

**Figure 4:**
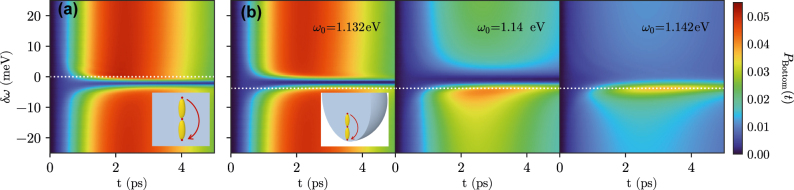
Population transfer to Bottom when Top and Bottom are fixed at a frequency *ω*_0_ and Gap is detuned from that frequency. The initial state *ψ*_0_ corresponds to only Top excited, as shown in both sketches in subplots (a) for the dielectric background and (b) for the dielectric sphere (shown in each subplot).

Next, we study energy transfer from the Top to the Bottom emitter, as depicted schematically in the insets of Figure 4. We use the same system and parameters as in the previous setup but now initialize the system in $|\psi_{0}〉=\sigma_{\text{Top}}^{+}|0〉$ and monitor the population $P_{\text{Bottom}}\left(t\right)=\left\langle \sigma_{\text{Bottom}}^{+}\sigma_{\text{Bottom}}^{-}\right\rangle$ . Panel (a) in Figure 4 again shows this for the purely plasmonic nanocavity. Population transferred to Bottom is then essentially unaffected by the presence of the Gap emitter unless that emitter is on resonance with the others (after taking into account the differing Lamb shifts). On resonance, energy transfer is significantly suppressed and the Gap emitter acts like an intermediate absorber. This simple picture is again mostly independent of *ω*_0_, so only a single value is shown in Figure 4(a). However, in the hybrid cavity, Figure 4(b), the picture changes drastically. In that case, the intermediate emitter can act to either enhance or suppress the population transfer depending on *ω*_0_ and *δω*, with a clear asymmetry with regard to the sign of *δω*. For *ω*_0_ = 1.14 eV, the maximum population reaching the Bottom emitter has a clear Fano-like interference shape with a maximum followed by a steep minimum as a function of *δω*. When the frequency of the Top and Bottom emitters is further increased by just 2 meV, to *ω*_0_ = 1.142 eV, the minimum essentially disappears and the presence of the Gap emitter on resonance leads to an enhancement of energy transfer. We thus find that within a narrow frequency range, the hybrid modes offer the possibility to change the role of the Gap emitter from inhibiting energy transfer between two emitters to enhancing it.

**Figure 5:**
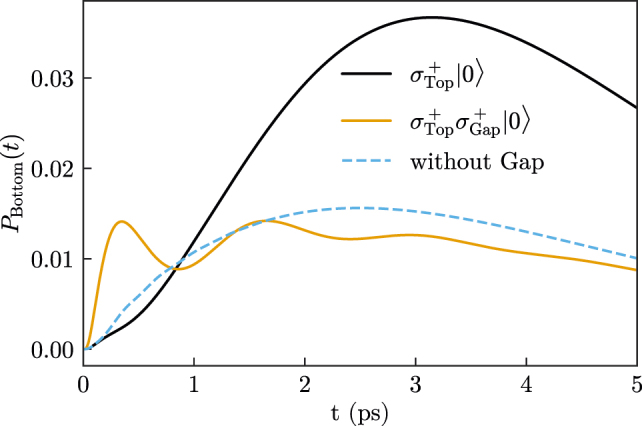
Population of Bottom for two-photon emission (black line) and one-photon emission (orange line) when Top and Bottom have frequencies *ω*_0_ = 1.142 eV and Gap is detuned *δω* = −3.78 meV.

Finally, we study how the energy transfer from the Top to the Bottom emitter changes when the Gap emitter is excited as well, i.e., for the initial state $|\psi_{0}〉=\sigma_{\text{Top}}^{+}\sigma_{\text{Gap}}^{+}|0〉$ . We here choose *ω*_0_ = 1.142 eV, corresponding to the final dataset in Figure 4(b), and choose the frequency of the Gap emitter so that in the single-excitation case studied previously, the population transfer to the Bottom emitter is maximized, achieved for *δω* = −3.18 meV. Figure 5 shows *P*_Bottom_(*t*) for the three cases where Gap is initially in its ground state (black line, same data as Figure 4), excited state (orange line), or is not present at all (dashed blue line). When the Gap emitter is initially excited, there is fast initial population transfer (presumably directly from the Gap to the Bottom emitter), but the maximum population reached is significantly lower than for the previous situation where energy transfer is enhanced by the presence of the (ground-state) Gap emitter. In particular, when the Gap emitter is initially excited, the maximum population in Bottom remains smaller than for the case where no emitter is present in the Gap. This shows that the energy transfer between the Top and Bottom emitters can be controlled by exciting the emitter in the Gap, pointing toward a possible path for implementing quantum gates based on hybrid metallodielectric structures.

## 4 Conclusions

In this article, we have introduced an extension of the few-mode field quantization approach we recently developed [1] to the case of several emitters. We have first demonstrated how to define and obtain the generalized spectral density J(ω) , a matrix-valued function that fully determines the properties of the EM environment interacting with the emitters. We have then shown how to obtain a few-mode quantization of the EM field in this situation. The resulting model can be adjusted to represent a wide range of nanophotonic structures by fitting the model parameters to reproduce the numerically calculated generalized spectral densities (which requires only the calculation of the dyadic Green’s function of Maxwell’s equations and can be done with any standard EM solver).

We illustrated the approach in a metallodielectric structure consisting of a metallic dimer embedded in a dielectric sphere, which produces a complex generalized spectral density, with *N* = 42 modes required to obtain a well-converged representation of the generalized spectral density. Once the fit is obtained, the emitter dynamics can be calculated using standard approaches of quantum optics such as solving the Lindblad master equation. We used this to study energy transfer between three emitters in three different situations and found that hybrid metallodielectric structures can enable significant control, strongly enhancing or suppressing energy transfer for slight variations of the emitter parameters.
